# Off-Axis and On-Axis Performance of Novel Acrylic Thermoplastic (Elium^®^) 3D Fibre-Reinforced Composites under Flexure Load

**DOI:** 10.3390/polym14112225

**Published:** 2022-05-30

**Authors:** Syed Zulfiqar Hussain Shah, Puteri S. M. Megat-Yusoff, Saravanan Karuppanan, Rizwan Saeed Choudhry, Zubair Sajid

**Affiliations:** 1 Mechanical Engineering Department, Universiti Teknologi PETRONAS, Seri Iskandar 32610, Perak, Malaysia; syedzulfiqar.shah@utp.edu.my (S.Z.H.S.); saravanan.karuppanan@utp.edu.my (S.K.); zubair_17005303@utp.edu.my (Z.S.); 2 School of Computing and Engineering, Mechanical Engineering Discipline, College of Science and Engineering, University of Derby, Derby DE22 1GB, UK; r.choudhry@derby.ac.uk

**Keywords:** 3D composites, thermoplastic, thermoset, off-axis flexure behaviour, on-axis flexure behaviour

## Abstract

The flexure response of novel thermoplastic (Elium^®^) 3D fibre-reinforced composites (FRC) was evaluated and compared with a conventional thermoset (Epolam^®^)-based 3D-FRC. Ten different types of sample 3D-FRC were prepared by varying fibre orientations, i.e., 0°, 30°, 45°, 60° and 90°, and resin system, i.e., thermoplastic and thermoset. The bending characteristics and failure mechanisms were determined by conducting a three-point bend test. Results elucidate that the on-axis specimens show linear response and brittle failure; in contrast, the off-axis specimens depicted highly nonlinear response and ductile failure. The thermoplastic on-axis specimen exhibited almost similar flexure strength; in comparison, the off-axis specimens show ~17% lower flexure strength compared to thermoset 3D-FRC. Thermoplastic 3D-FRC shows ~40% higher energy absorption, ~23% lower flexure modulus and ~27% higher flexure strains as compared to its thermoset counterpart.

## 1. Introduction

The use of 3D-FRC has increased in recent years thanks to their superior transverse properties (impact resistance) and damage tolerance in comparison with 2D-FRC [1,2,3,4,5]. However, their in-plane properties were reduced, which was attributed to the waviness caused by the z-binder [6,7,8,9]. Following this direction, researchers focused their attention on improving their in-plane properties [10,11,12]. Meanwhile, their failure mechanisms and bending behaviour are still unclear [13]. The 3D-FRCs are orthotropic, and their load-carrying capacity varies with the fibre orientation. In addition to fibre orientation, resin toughness significantly affects bending characteristics. Therefore, it is desirable to understand the effect of fibre orientation and resin toughness on the flexure properties of 3D-FRC for their design and different applications.

The thermoset polymers took the lead as a matrix for FRC due to their ease in manufacturing processes in comparison with thermoplastic polymers. The thermoplastic FRC offers attractive advantages, i.e., higher impact resistance, damage tolerance and the ability to be recycled at the end of life [14,15,16]. However, the major roadblock is the manufacture of thermoplastic FRC, as the viscosity of the thermoplastic matrix in its molten state is high. In particular, the manufacture of 3D-FRC with thermoplastic is challenging due to poor impregnation [17]. Currently, post-impregnation is generally used to manufacture 3D-FRC, as long as the thermoplastic polymers are in the form of powder/fibre or film [18]. This limits the manufacture of 3D-FRC with thermoset matrix using conventional manufacturing processes, i.e., resin infusion and resin transfer moulding [19,20].

In past years, the flexure properties of thermoset-based 3D-FRC were widely studied [21]. Several researchers studied their on-axis (warp and fill) flexure properties [22,23,24,25,26] and concluded that delamination resistance and bending properties were improved by introducing a z-binder in the fabric architecture [27,28]. Apart from the on-axis flexure properties, the off-axis bending behaviour of 3D-FRC was rarely discussed. Recently, Zhang et al. [13] studied the on-axis (0° and 90°) off-axis (30° and 45°) flexure behaviour of 3D-FRC and found that the on-axis specimen shows a brittle response compared to off-axis specimens. All the studies discussed above were limited to thermoset-based 3D-FRC. Very few researchers discussed the flexure properties of thermoplastic-based 3D-FRC [17,18,28,29]. The flexure modulus of thermoplastic FRC is generally lower than thermoset FRC, which was attributed to the higher toughness of the thermoplastic matrix. Kuo et al. [18] studied the flexure properties of polyamide-based 3D-FRC and reported a reduction in fibre kinking. Qian et al. [28] studied polypropylene-based 3D-FRC and found that the flexure properties improved. Both studies highlight an improper impregnation of 3D fabric due to the higher viscosity of polypropylene and Polyamide resin. Archer et al. [29] compared the flexure properties of thermoset (epoxy) and thermoplastic (polybutylene terephthalate) based on 3D-FRC. They observed that the flexure strength of thermoplastic-based 3D-FRC was 46% less than that of thermoset 3D-FRC due to the highly crystalline microstructure and voids produced during the fabrication process. Luo et al. [17] investigated PEEK based on 3D-FRC and reported an improper impregnation of yarns. Moreover, they used 3D fabric with a small thickness (2 mm) and low fibre volume fraction (36%). Hence, manufacturing 3D-FRC with higher thickness and fibre volume fraction using post-impregnation process (such as hot compression molding) is again a challenging task.

To address these limitations, Arkema recently developed acrylic thermoplastic resin (Elium), which is in liquid form at room temperature and can be employed to manufacture thermoplastic 3D-FRC by using conventional manufacturing processes, i.e., vacuum infusion. Elium^®^ has tremendous potential for a wide range of applications in composite industries such as aerospace, automotive, wind power, sports and marine industries, etc. In particular, it is beneficial for the wind power industry where it can be used to manufacture wind turbine blades using a conventional resin-assisted vacuum infusion process. These wind turbine blades will not only provide better fatigue life and resistance against the extreme environment but also resolve the recycling problem at the end of their life span. Furthermore, it offers a unique advantage to assembling composite parts by using resistance or ultrasonic welding, which are particularly significant for aerospace, wind power and automotive industries [30].

Recent studies highlighted that the novel resin-infused thermoplastic-based 3D-FRC is a suitable material to manufacture composite structures for superior impact resistance [2,31], damage tolerance [32] and energy absorption [33] applications. Murray et al. [34] studied the flexure properties of a novel acrylic thermoplastic-based 2D-FRC and reported that the flexure strength and flexure modulus were almost similar. To the best of the author’s knowledge, no study has been dedicated to evaluating the flexure performance of novel acrylic thermoplastic-based 3D-FRC and their comparison with thermoset-based 3D-FRC. Therefore, it is important to evaluate the bending characteristics and delamination resistance of this novel acrylic resin (Elium^®^)-based 3D-FRC before they are considered for practical applications. 

Following these directions, the objective of this paper is to determine the combined effect of fibre orientation (on-axis/off-axis) and matrix toughness (Elium^®^ and Epolam^®^) on the flexure properties of 3D-FRC. Ten different types of 3D-FRC samples with different fibre orientations (0°, 30°, 45°, 60° and 90°) and matrix (Elium^®^ and Epolam^®^) were tested using a three-point bend test. For both thermoplastic and thermoset 3D-FRC, only one fibre volume fraction has been investigated. The results were compared in terms of flexure strength, flexure modulus, flexure strain and stress-deflection curves. Moreover, a detailed macroscopic and microscopic damage characterization was performed using digital and Scanning Microscopy (SEM) images to identify the effect of both factors on the damage mechanisms of 3D-FRC under flexure load.

### 1.1. Flexure Response of FRC

**Figure 1:**
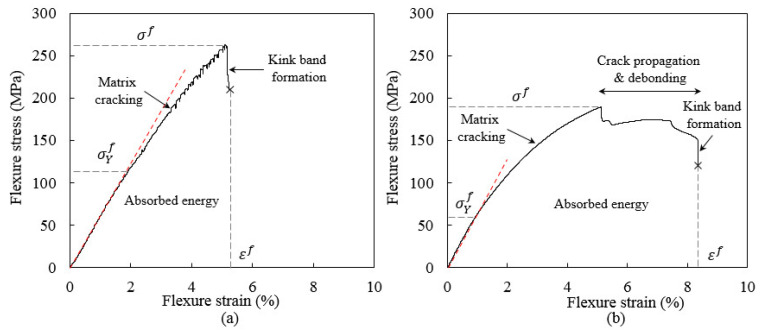
Schematic diagram of typical flexural stress/strain curve of 3D-FRC: (**a**) on-axis S-D curves of 3D-FRC and (**b**) off-axis stress/displacement curves of 3D-FRC.

The flexure response of 3D-FRC depends on the fabric architecture, fibre orientation (on-axis and off-axis), resin toughness (thermoplastic and thermoset) and tensile/compressive properties. Under flexure load, the top surface of the specimen experiences compressive load, whereas the bottom surface undergoes tensile load [35]. Figure 1. shows the schematic diagram of the stress–strain curve under flexural load, which provides a complete failure response of 3D-FRC (damage initiation and propagation). Figure 1a depict the on-axis flexural stress-strain curve of 3D-FRC. The flexure load increases linearly until it reaches the maximum value, which is called flexure yield stress “$\sigma_{Y}^{f}$”. As the load increases further, the nonlinear region begins and the flexure modulus decreases due to fibre kinking (at/near the top surface) or matrix cracking (at the bottom surface). During this process, the stress reached its ultimate value called ultimate flexure stress “$\sigma^{f}$”. After this point, the transverse crack on the bottom surface propagates upward and transfers all loads to yarns/fibres, which results in their failure. This fibre/yarn breakage produces a sudden load drop (~20%) in the stress–strain curve as a result of kink band formation, which is the strongest limiting factor. The strain at this point is called flexural strains “$\varepsilon^{f}$”. Figure 1b depicts the off-axis flexural response of 3D-FRC. After reaching the ultimate flexure stress, the damage propagates in the form of crack extension, debonding and fibre kinking. During this stage, no catastrophic failure occurs, and the load-bearing capacity of a material decreases. The stress–strain curve shows a large plateau during this stage due to geometric deformation and re-orientation of yarns, which is also called the “scissoring effect” [13]. This large plateau indicates that the off-axis specimen absorbs more energy and exhibited significant high impact resistance and damage tolerance.

## 2. Materials and Methods

### 2.1. Material Used

**Figure 2:**
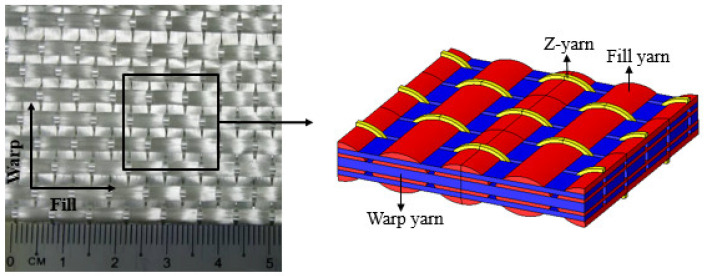
3D orthogonal woven fabric (3D-9871).

The 3D fabric used in this study is orthogonal E-glass woven fabric (3D-9871) obtained from TexTech^®^ Industries, Winthrop, ME, USA. The actual fabric and schematic diagrams of 3D orthogonal woven fabric are depicted in Figure 2. The areal weight of the fabric is 5200 g/m^2^, and the overall thickness of a single layer is ~4 mm. In this study, the 3D-FRC panels were fabricated using both thermoplastic and thermoset resin systems, i.e., a recently developed acrylic thermoplastic liquid resin Elium^®^ 188x0 from Arkema, Colombes, France, and thermoset epoxy resin system Epolam^®^ 5015/5015 from Axson, Shanghai, China. Elium^®^ 188x0 is an acrylic monomer that was mixed with a peroxide initiator to initiate the polymerization process at room temperature. The mechanical properties of both resin systems are summarised in Table 1. 

### 2.2. Fabrication Process

**Figure 3:**
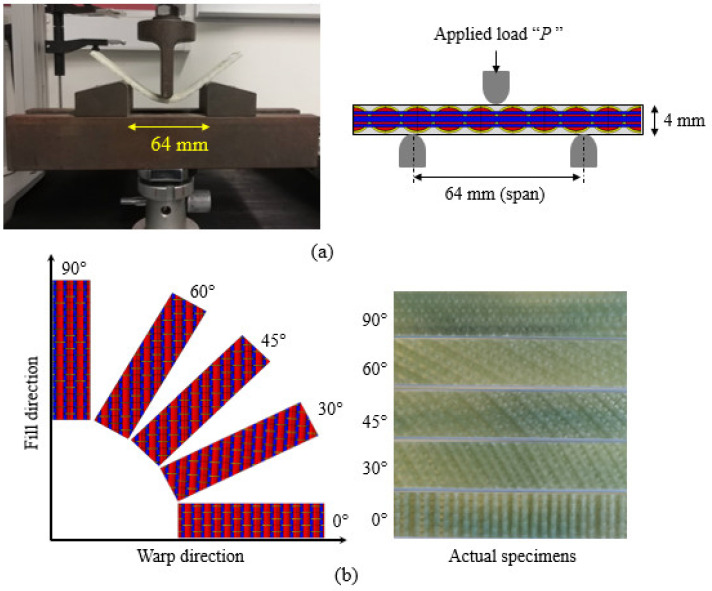
Samples prepared for the flexure testing of 3D-FRC: (**a**) 3D fabric architecture with different fibre orientations and actual specimens cut into different orientations and (**b**) flexure testing setup and schematic diagram of three-point bend test.

Elium^®^ and Epolam^®^ based 3D-FRCs were manufactured by using a vacuum-assisted resin transfer moulding (VARTM) process. The mixed viscosity of Elium^®^ 188x0 and Epolam^®^ 5015 are 200 mPa.s and 210 mPa.s, respectively, which is ideal for the VARTM process. A rectangular panel of 400 mm × 500 mm was fabricated. The resin systems were mixed carefully for two-three minutes to obtain a homogenous mixture. In the case of the Elium^®^ based 3D-FRC, resin was degassed for 15 min due to its short pot life (60–70 min). The resin infusion was carried out at 100 mbar to avoid resin boiling and the process was completed in 25 min. The panels were left at room temperature to complete the polymerization process, followed by post-curing in an oven at 80 °C for four hours to achieve maximum mechanical properties. In contrast, for the manufacture of Epolam^®^-based 3D-FRC, Epolam^®^ resin was degassed for 25 min to remove air bubbles, which may degrade the final mechanical properties. The resin infusion was performed at 450 mbar and completed in 7 min. Epolam^®^-based 3D-FRC panels were left for twelve hours to fully cure at room temperature and then post-cured in an oven at 80 °C for eight hours. Figure 3 also shows a comparison of infusion time in the Elium^®^ and Epolam^®^-based FRCs. The Elium^®^ based 3D-FRC took approximately fourfold to completely infuse the panel because low vacuum pressure was used to avoid void/bubble formation during the polymerization process. More details on the fabrication process can be found in our earlier publications [6,31,32,39].

### 2.3. Physical Parameters of the Cured Panels and Samples

After the panels were fully cured, they were checked for defected regions, and physical parameters such as density, fibre volume fraction and thickness of the panel were measured. The fibre volume fraction of fabricated panels was measured using the burn-off method according to ASTM D3171, and density was measured using the water displacement method according to ASTM D792-08. Ten samples were cut from different panels, and the average void content, density and fibre volume fractions were measured. In Elium^®^-based 3D-FRC, the void content was less than 3.7%; in comparison, in Epolam^®^-based 3D-FRC, the void content was less than 1%. The fibre volume fraction of Elium^®^ and Epolam^®^-based 3D-FRC are 52 ± 1.5% and 52 ± 0.4%, respectively. The thickness of the cured panels was measured at different locations, and the average values are 4 ± 0.05 mm. The densities of the cured Elium^®^ and Epolam^®^-based 3D-FRC were 1.86 g/cc and 1.92 g/cc, respectively, and they were measured using the water displacement method. 

### 2.4. Flexural Testing

The flexure test was performed according to ASTM standard D7264 (procedure A, i.e., three-point bend test), which requires a rectangular specimen [40]. The specimen rests on two vertical supports, while the load was applied halfway between vertical supports, as shown in Figure 3a. The span to thickness ratio used was 1:16 as recommended by the standard. The total length of the specimen should be 10% longer than the span length from each side of the vertical support. The final dimensions of a rectangular specimen were 100 mm × 25 mm × 4 mm. The load rate used was 2 mm/min. Three samples were tested for both 3D-FRC materials at each orientation. The properties measured from the flexure test were flexure strength, modulus and deflection. The flexure strength and modulus can be calculated using Equations (1) and (2), respectively:

(1) $$\sigma_{f}=\frac{3FL}{2bd^{2}}$$

(2) $$E_{f}=\frac{L^{3}}{4bd^{3}}\frac{\Delta P}{\Delta \delta }$$

where “*L*”, “*b*”, “*d*” and “*F*”, represent support span, specimen width, specimen thickness and applied load, respectively. “∆*P*” and “∆*δ*” represent load increments and deflection increments, respectively. In this study, thirty samples were prepared (three samples for each fibre orientation, i.e., 0°, 30°, 45°, 60° and 90°) for both materials (Elium^®^ and Epolam^®^ based 3D-FRC). Figure 3b shows the 3D fabric architecture, fibre orientation in each sample and actual sample prepared for testing. The specimens were cut from panels using a diamond tip disc cutter, which provides an excellent surface finish.

### 2.5. Damage Evaluation Method

To further understand the failure mechanisms in both 3D composites, a comprehensive fractography was performed. The failure mechanisms were classified into intralaminar and interlaminar failure mechanisms. A collection of digital photographs obtained following mechanical tests was used to examine the macro-damage morphologies of failed specimens. Meanwhile, SEM was used to examine the micro-failure processes. Because both types of 3D composites were made with insulating glass fibres, the specimens’ surfaces were covered with a 40 nm gold layer to make them conductive. The voltage and maximum magnification used during SEM analysis are 10 keV and 200×, respectively.

## 3. Results and Discussion

### 3.1. Comparison of the Stress-Displacement Curve

**Figure 4:**
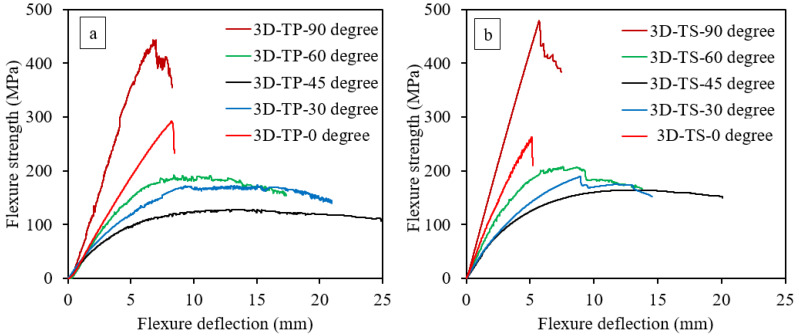
Effect of fibre orientation on the flexure properties of 3D-FRC, (**a**) S-D curves of 3D thermoplastic FRC and (**b**) S-D curves of 3D thermoset FRC.

Figure 4 depict the flexure stress-deflection curves of Elium^®^ and Epolam^®^ based 3D-FRC. The stress-displacement curve provides comprehensive details about the damage process of 3D-FRC under flexure load, as discussed in Section 1.1. Each stress-displacement curve in Figure 4 represents different fibre orientations and matrix systems, which highlight that flexure properties were sensitive to both matrix toughness and fibre orientation. All stress-displacement curves show linear behaviour initially, followed by non-linear behaviour after 1% to 1.5% of flexure deflection.

The on-axis specimens (0° and 90°) show higher bending stiffness and load-bearing capacity than the off-axis specimen (30°, 45° and 60°). It was observed that on-axis specimens failed in a brittle manner (that ism with a sudden fast fracture and little signs of progressive damage or plastic deformation as compared to off-axis specimens). The stress-displacement curve of on-axis specimens shows linear behaviour up to a maximum value (ultimate strength), followed by the sudden load drop caused by the tensile fibre failure at the bottom surface of the specimen. Such brittle behaviour of on-axis specimens is due to fibre-dominant characteristics, and a similar observation was made by Zhang et al. [13]. This sudden failure of the on-axis specimen may represent structure damage and is completely lost in the load-bearing capacity of the material. In comparison, the off-axis specimen shows a plateau in the stress–strain curve analogous to the plastic plateau observed in ductile metals. In this case, the plateau is formed due to the progressive failure of the matrix and fibres, which results in a larger strain to final failure, bending deflection, lower bending stiffness and lower peak loads compared to the on-axis specimens. This large deflection represents the matrix dominant characteristics of off-axis specimens, which is consistent with references [13,41]. The ranking for the maximum flexure strength at different fibre orientation is as follows: 90° > 0° > 60° > 30° > 45°. This is consistent for both types of 3D-FRC.

Regarding the effect of matrix toughness on flexural properties, the thermoplastic 3D-FRC on-axis specimen shows almost similar ultimate flexural strength; in contrast, the off-axis specimens depict lower ultimate stress as compared to the thermoset counterpart (see Figure 4). The bending stiffness of thermoplastic 3D-FRC was lower, whereas the flexure deflection is higher due to the plastic deformation of a ductile matrix (see Figure 4a). The thermoset 3D-FRC specimens (30° and 60°) undergo a sudden load drop, as shown in Figure 4b. This is postulated to be due to kinking and yarn/matrix interface debonding. In contrast, the thermoplastic 3D-FRC specimens (30° and 60°) continue to deform without catastrophic damage, as shown in Figure 4a. This behaviour of thermoplastic 3D-FRC is due to their higher interlaminar fracture toughness, which delays crack propagation [29]. Moreover, the thermoplastic 3D-FRC specimen (45°) shows the highest bending deflection and lowest peak load. The ranking for the maximum flexure modulus at different fibre orientations is the same as flexure strength, i.e., 90° > 0° > 60° > 30° > 45°.

### 3.2. Comparison of Flexure Strength, Modulus and Failure Strain

The results obtained from flexure tests in terms of modulus, ultimate stress, yield stress and strains were summarised in Table 2. The values in the parenthesis represent the coefficient of variance (COV) among three tested specimens for each configuration. The on-axis specimens show higher COV due to fibre dominant characteristics and sensitivity to the geometric variabilities. In contrast, the off-axis specimens depict less variation, which was attributed to the progressive nonlinear damage due to the reorientation of yarns. 

The results depict that the on-axis specimens show the highest flexure strength in both 3D-FRCs. The flexural strengths of Elium^®^ and Epolam^®^-based 3D-FRC were 249 MPa and 225 MPa when loaded along the warp direction and 418 MPa and 455 MPa when loaded along fill direction, respectively. The off-axis specimens experience the lowest bending strength due to the in-plane shear induced in these composites, which manifests itself in the re-orientation of angled yarns (warp and fill) towards the principal directions. This has been referred to in the literature as the scissoring effect [13,42]. During this re-orientation process, the specimen experiences additional flexural strains due to geometrical deformation, which is followed by yarn/matrix debonding. Both these phenomena are responsible for the large deflection of the specimen under flexure loads. Among the off-axis specimens, the 45° fibre orientation specimen depicts the lowest flexure strength (130–170 MPa), modulus (5.4–6.5 GPa) and strain (11–14%). 

**Figure 5:**
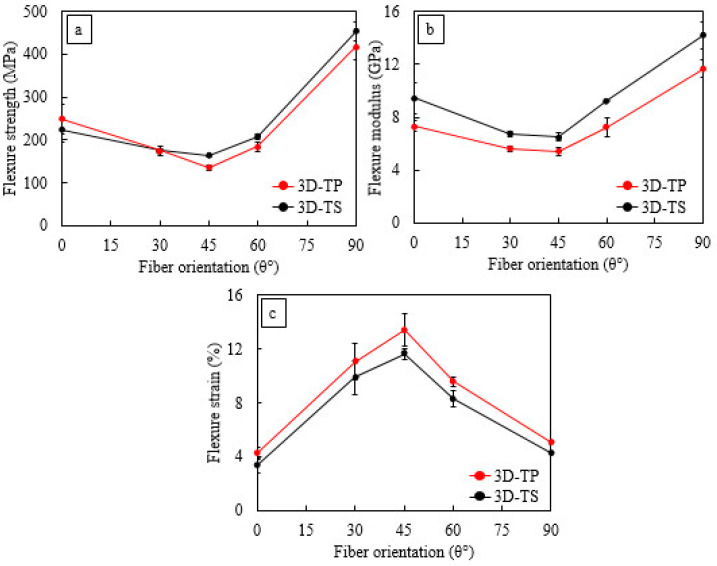
Variation of flexure strength, modulus and failure strain as a function of fibre orientation in 3D-FRC. (**a**) Flexural strength, (**b**) Flexural modulus and (**c**) Flexural strain.

In order to evaluate the effect of resin toughness on the flexure properties of 3D-FRC, the flexural strength, modulus and strain were compared, as shown in Figure 5. The thermoplastic 3D-FRC possesses lower ultimate flexural stress compared to thermoset 3D-FRC except at 0° and 30°, whereas the thermoplastic composite shows higher ultimate stress, as shown in Figure 5a. The warp-loaded thermoplastic and thermoset composite specimens exhibited 40% and 50% lower ultimate flexural stress, as compared to fill-loaded specimens, respectively. One possible reason for this lower stress is the difference in the fabric architecture in both directions. In warp-loaded specimens, the resin-rich pockets at the top/bottom surface were parallel to the applied load. Under flexural load, the cracks developed in the brittle Epolam^®^ matrix propagate more rapidly, leading to the earlier failure at lower ultimate stress. In contrast, the ductile thermoplastic matrix undergoes plastic deformation and delays the propagation of cracks, which results in relatively higher ultimate stress along the warp direction. 

Figure 5b shows the comparison of flexural modulus. The flexure modulus of Elium^®^-based 3D-FRC was 16–29% lower than the thermoset counterpart. The 3D fabric used in the study exhibited the same areal density along the warp and fill direction; theoretically, the flexure modulus should be the same. However, it is worth noting that the flexural moduli of warp-loaded thermoplastic and thermoset composites were 38% and 34% lower than fill-loaded specimens, respectively. A probable explanation of the lower flexure modulus is that the middle yarn warps as it runs through the neutral axis of the specimen. The cross-sectional area of the middle warp yarn is twice as large as compared to the top/bottom warp yarn. Thus, 50% of the main load-carrying yarn was not fully utilised/loaded under flexure load. Figure 5c depicts the comparison of flexure strains. Thermoplastic 3D-FRC possesses 15–30% higher flexure strains due to the ductile matrix and higher fracture toughness. In comparison, the brittle Epolam^®^ matrix failed earlier due to the rapid propagation of micro-cracks, which led to an early failure of 3D-FRC. The ranking for the maximum flexure strain at different fibre orientations is almost opposite to the flexure strength and modulus, i.e., 45° > 30° > 60° > 90° > 0°. This ranking is the same for both types of 3D-FRC. 

**Figure 6:**
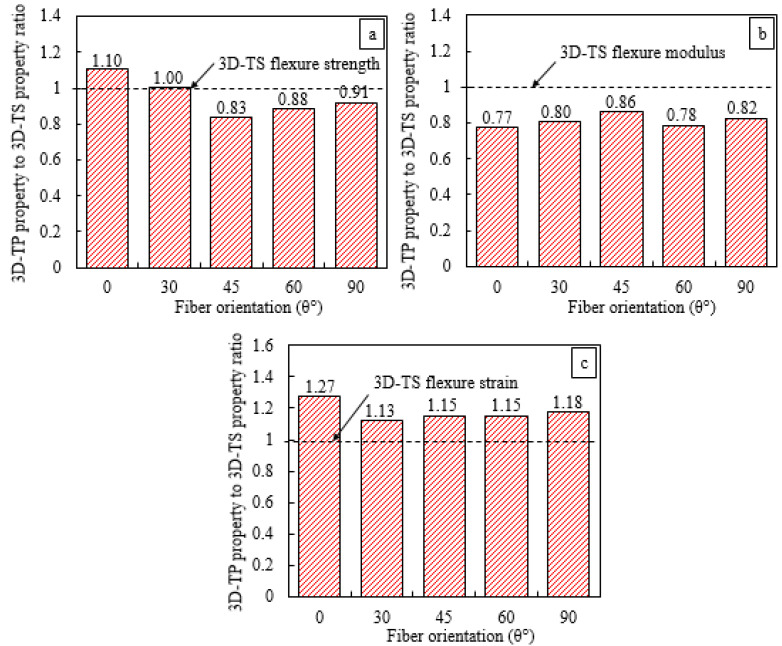
Comparison of normalised flexure strength, modulus and failure strain of 3D-FRC. (**a**) Flexural strength, (**b**) Flexural modulus and (**c**) Flexural strain.

Normalizing the flexure properties of the thermoset 3D-FRC with thermoplastic 3D-FRC is one possible comparison to evaluate the performance of both materials when undergoing different loadings. Moreover, it clearly highlights the performance of both 3D-FRC in terms of flexure strength, modulus and strain at maximum stress, as shown in Figure 6. The visual comparison clearly indicates that the ultimate flexural stress of on-axis novel Elium^®^-based 3D-FRC was only 9% lower (see Figure 6a), as compared to 46% lower ultimate stress as reported by Archer et al. [29]. The flexure modulus of Elium^®^ based 3D-FRC was ~23% lower, as shown in Figure 6b. In comparison, the flexure strain of Elium^®^-based 3D-FRC was ~27% higher, as shown in Figure 6c.

### 3.3. Flexural vs. Yield Strength of 3D-FRC

**Figure 7:**
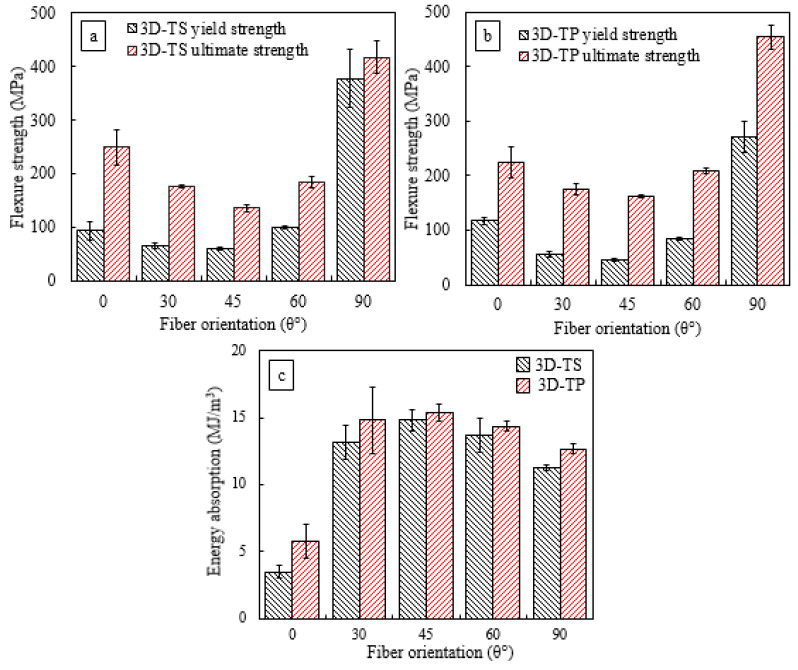
Comparison of flexure strength and flexure yield strength as a function of fibre orientation, (**a**) 3D thermoplastic FRC, (**b**) 3D thermoset FRC and (**c**) comparison of energy absorbed by 3D-FRC under flexure load.

Figure 7 depicts the comparison between ultimate flexure stress and flexural yield stress as a function of fibre orientation. The error bars represent the variation in the data. The flexure yield stress in the stress–displacement curve represents a point where the curve lost its linearity, as discussed in Section 1.1. Overall, the thermoplastic 3D-FRC possesses lower flexural yield stress compared to the thermoset counterpart, as shown in Figure 7a,b. The flexural yield stresses of fill loaded thermoset and thermoplastic-based 3D-FRC were 82% and 65% of the ultimate flexure stress. In comparison, the flexural yield stresses of warp loaded Epolam^®^ and Elium^®^-based 3D-FRCs were 42% and 45% of ultimate yield stress. This lower yield stress along the warp direction is due to the resin-rich pockets on the top/bottom surface of the specimens. Matrix cracks developed in these resin pockets grew rapidly, which leads to lower flexure yield stress. In the case of off-axis specimens, the flexure yield stress varies between 30 and 50% of the ultimate flexure stress.

### 3.4. Effect of Resin Toughness on Energy Absorption

Figure 7c shows the comparison of energy absorbed by 3D-FRC per unit volume (MJ/m^3^), which was calculated through the area under the stress/strain curve, up to complete failure. Overall, Elium^®^-based 3D-FRC absorbed up to 40% higher energy compared to Epolam^®^-based 3D-FRC. The on-axis specimens absorbed less energy compared to off-axis specimens due to fibre-dominant characteristics. This higher energy absorption of off-axis specimens was due to the scissoring effect, as discussed earlier. The off-axis thermoplastic 3D-FRC absorbs almost two-times higher energy compared to on-axis specimens. In comparison, the off-axis thermoset 3D-FRC absorbs around three-times higher energy than on-axis specimens, which was attributed to the sudden brittle failure of on-axis specimens at much lower flexure deflections. The above findings indicate the following: (i) the thermoplastic 3D-FRC exhibited much better energy absorption capability; (ii) the off-axis specimens are the better choice for composite structures, where higher energy absorption is the prime requirement.

### 3.5. Failure Mechanisms in TP and TS 3D Composites under Flexure Load

**Figure 8:**
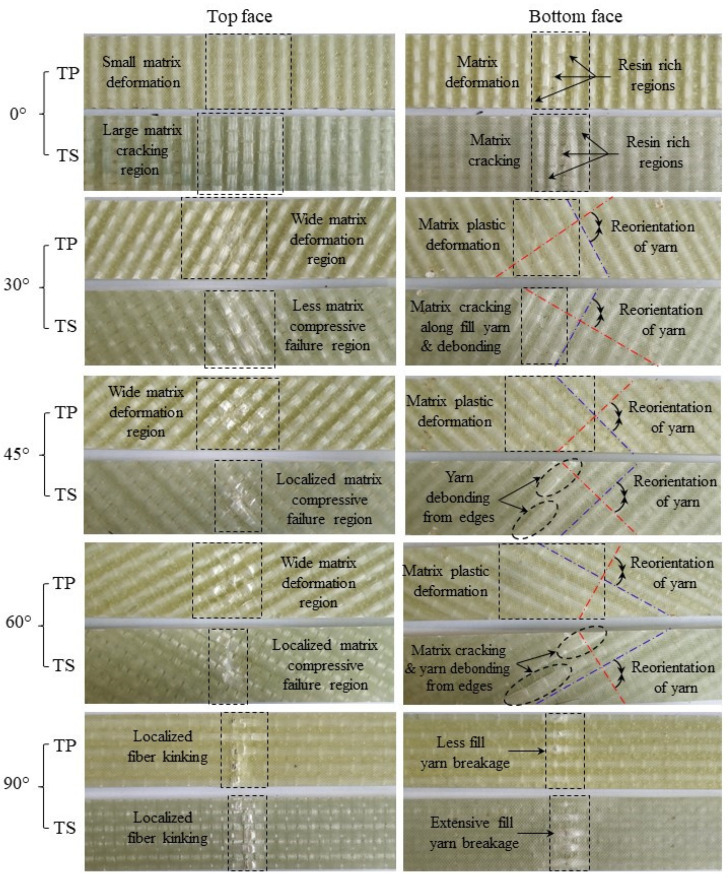
Comparison of macroscopic damage patterns in 3D-FRC. Blue-line represents fill yarn, and the red line represents warp yarn.

The failure mechanisms in 3D-FRC under flexural loads have been shown in Figure 8. The figure shows the combination of macro- and micro-failure mechanisms. The main damage mechanisms in the thermoset 3D-FRC were yarn kinking, yarn tensile failure, matrix cracking, debonding/delamination and re-orientation of yarns. In comparison, the failure mechanisms in thermoplastic-3D-FRC were yarn kinking, yarn tensile failure, matrix plastic deformation and re-orientation of yarns. The bottom surface of the specimen experiences the highest damage due to tensile stress developed under flexural loads. The presence of various failure mechanisms made it challenging to evaluate damage mechanisms by changing fibre orientation under flexural loads. Therefore, the failure mechanisms were divided into two main recognised categories, i.e., intralaminar failure mechanisms (micro and macro damages of fibre and matrix in impregnated yarn and matrix regions) and interlaminar failure mechanisms (delamination as well as debonding due to reorientation of yarns). These failure mechanisms for all cases have been summarised in Table 3 and are discussed in the following section.

#### 3.5.1. Intralaminar Failure Mechanisms

**Figure 9:**
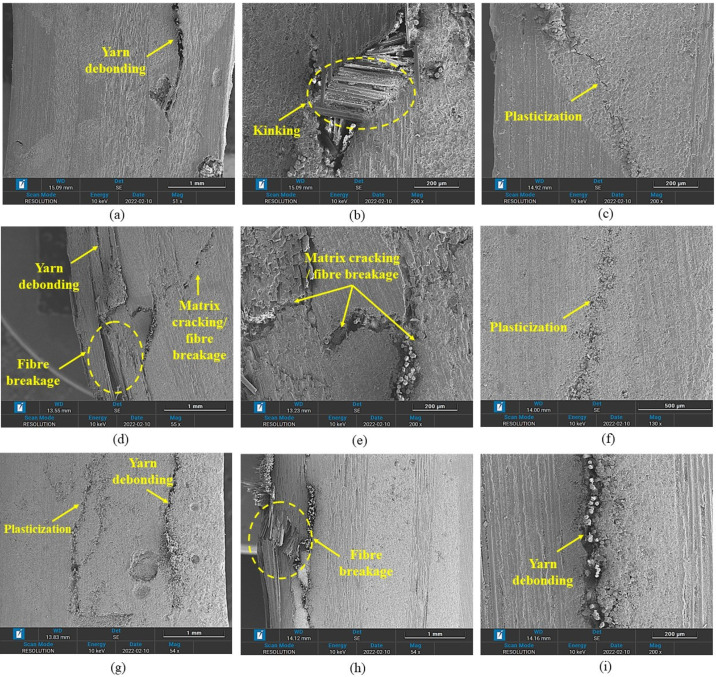
Comparison of intralaminar/interlaminar failure mechanisms in on-axis (0º and 90º) specimens. (**a**–**c**) 0º TP-3D-FRC, (**d**,**e**) 0º TS-3D-FRC, (**f**,**g**) 90º TP-3D-FRC and (**h**,**i**) 90º TS-3D-FRC.

The behaviour of 3D-FRC, when loaded along the on-axis direction, can be described in a general sense as linear, elastic and brittle due to straight yarns, which carry the majority of the load and allow for little deformation before catastrophic failure (see Figure 4). The on-axis specimens (0° and 90°) show localised damage zone on the top and bottom surface, due to fibre breakage in warp or fill yarns (see Figure 9). In the case of warp-loaded specimens (0°), the resin-rich pockets between the fill yarn undergo micro-cracking or elastoplastic deformation, which transfers all the load to warp yarn. In thermoset 3D-FRC, this micro-crack propagates rapidly, which results in catastrophic failure in the form of fibre breakage (see Figure 9d,e) at a lower deflection. On the other hand, the resin-rich pockets in thermoplastic 3D-FRC deform instead of cracking and delay crack propagation in the form of yarn debonding and kinking (see Figure 9a–c). Hence, these composites failed at a relatively higher flexural strain. In the case of fill-loaded specimens (90°), the fill yarns fail in compression due to kink band formation on the top surface and tensile failure at the bottom surface (see Figure 9f,g). However, in thermoset 3D-FRC, extensive fill yarn failure and yarn debonding were observed on the top and bottom surface due to the brittle matrix (see Figure 9h,i). 

**Figure 10:**
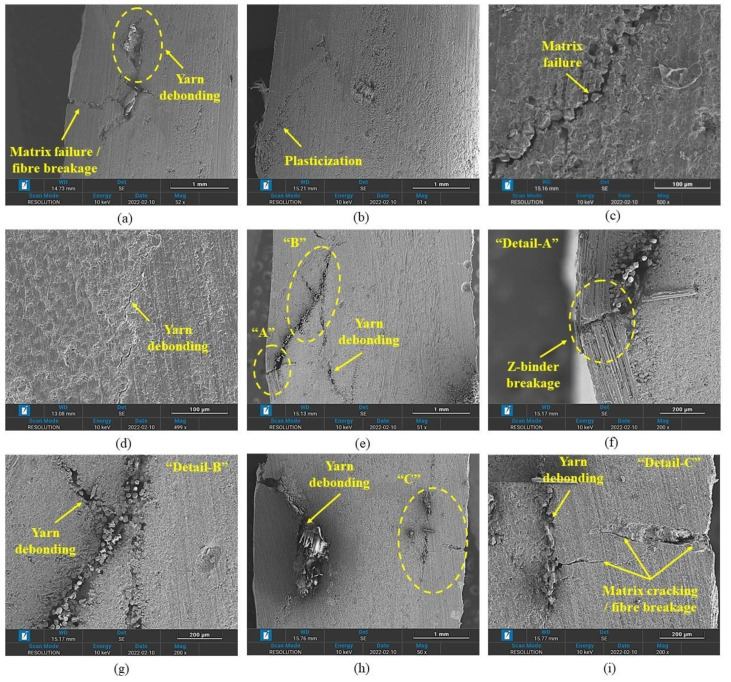
Comparison of intralaminar/interlaminar failure mechanisms in off-axis (30º, 45º and 90º) specimens. (**a**–**d**) TP-3D-FRC and (**e**–**i**) TS-3D-FRC.

The off-axis specimens (30°, 45° and 60°) undergo large deflection under bending loads, which results in higher damage. Among the off-axis specimens, the 45° specimens show the highest damage at the top and bottom face (see Figure 8). In comparison, the 30° specimen depicts the highest matrix compressive failure zone at the top surface, whereas the 60° specimens show the highest damage at the bottom surface. The thermoplastic off-axis specimens show a wider matrix compressive failure region on the top surface due to the ductile matrix (see Figure 8) and yarn debonding (see Figure 10d–f), whereas the bottom surface undergoes large plasticization (see Figure 10a,b). In comparison, thermoset 3D-FRC depicts a relatively small matrix compressive failure region (see Figure 8) and matrix cracking (see Figure 10h) on the top surface due to the brittle (Epolam^®^) matrix. In contrast, the bottom surface of the specimen undergoes extensive matrix cracking, yarn debonding and z-binder breakage, as shown in Figure 10e,h.

#### 3.5.2. Interlaminar Failure Mechanisms

**Figure 11:**
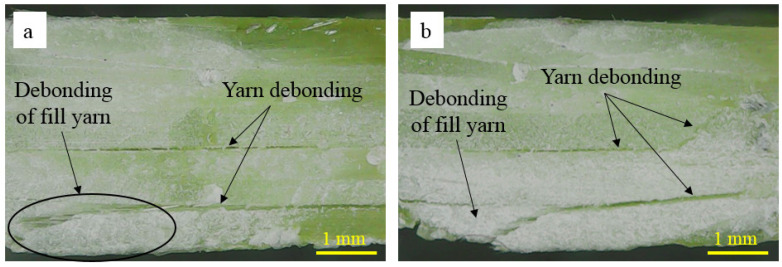
Interlaminar failure in off-axis specimens under flexure load. (**a**) Thermoplastic 3D-FRC and (**b**) thermoset 3D-FRC.

The off-axis behaviour of the 3D orthogonal woven composite is highly nonlinear due to the plasticization of resin-rich pockets and progressive damage of the matrix, which was attributed to the re-orientation of yarns. During the re-orientation process, the debonding/delamination of yarns and matrix occurs (see Figure 9 and Figure 10). The re-orientation between warp and fill yarns is shown in Figure 8. (Represented with blue and red lines). Moreover, Elium^®^-based 3D-FRC shows significantly reduced delamination (see Figure 9f,g) due to their higher interlaminar fracture toughness and strong fibre/matrix interface [43]. In contrast, extensive fill yarn debonding was identified in the Epolam^®^-based 3D-FRC due to the re-orientation of warp and fill yarns (Figure 9h,i). The off-axis specimens (45° and 60°) show significant debonding of fill yarns at the edges of the specimens in both 3D textile composites, as shown in Figure 11. Thermoplastic off-axis specimens show large out-of-plane permanent deformation after unloading due to the locking of yarns caused by plasticization during the loading process. 

This study elucidates that the novel thermoplastic 3D-FRC exhibited significantly improved bending properties as compared to conventional Elium^®^ based 3D-FRC. Although the bending properties of novel thermoplastic 3D-FRC were almost similar to the thermoset counterparts, it provides an additional advantage of superior impact resistance, damage tolerance and recyclability at the end of service life.

## 4. Conclusions

This work presents the experimental investigation on the effect of resin toughness and fibre orientation on the flexure properties of 3D-FRC. The on-axis specimens show the highest flexure strength and modulus due to brittle response, whereas the off-axis specimens undergo higher flexural strains, which were attributed to ductile characteristics. The thermoplastic on-axis 3D-FRC exhibited almost similar flexure strength, ~23% lower flexural modulus and ~27% higher flexural strains compared to thermoset 3D-FRC. In comparison, thermoplastic off-axis 3D-FRC demonstrated ~17% lower flexural strength, ~22% lower flexural modulus and ~15% lower flexural strains. On the other hand, in terms of energy absorption, thermoplastic 3D-FRC absorbs up to ~40% higher energy compared to the thermoset counterpart. The main damage mechanism in the thermoset 3D-FRC was matrix cracking, fill yarn failure and a significant delamination of fill yarn. In comparison, thermoplastic 3D-FRC shows plasticization, fill yarn failure and slight delamination. This improved performance of novel thermoplastic composites was attributed to strong fibre/matrix interface properties and higher interlaminar fracture toughness. This study highlights that the novel Elium^®^ matrix improves the bending properties of 3D-FRC compared to the conventional thermoplastic matrix. Hence, this novel thermoplastic 3D-FRC can be used in different applications, where higher flexure properties and energy absorptions are required.

## Figures and Tables

**Table 1:** Summary of mechanical properties of the thermoplastic matrix (Elium^®^ 188x0) and thermoset matrix (Epolam^®^ 5015).

Property	Elium^®^ 188x0	Epolam^®^ 5015/5015
Tensile strength (MPa) ^a^	76	80
Tensile modulus (GPa) ^a^	3.3	3.1
Elongation at failure (%) ^c^	6	3.1
Flexural strength (MPa) ^a^	130	100
Flexural modulus (GPa) ^a^	3.25	2.6
Fracture toughness (kJ/m^2^) ^b^	0.5	0.12
Rockwell Hardness ^d^	99	119
Density (g/cc) ^d^	1.17	1.15

^a^ Material technical datasheet. ^b^ Reported in the literature [36]. ^c^ Reported in the literature (Elium^®^ 188x0 [37] and Epolam^®^ 5015/5015 [38]). ^d^ In-house testing.

**Table 2:** Summary of flexure test results. Value in the parenthesis represents the co-efficient of variance in data.

Material	Fibre Orientation (Degree)	Flexure Modulus (GPa)	Flexure Yield Strength (MPa)	Flexure Strength (MPa)	Flexure Strain (%)
3D-TP-FRC	0°	7.3 (6.1%)	117 (14.5%)	249 (15%)	4.3 (12%)
	30°	5.6 (4.7%)	55 (9.0%)	176 (1.9%)	11 (12%)
	45°	5.4 (6.6%)	45 (4.5%)	136 (6.0%)	13.4 (9.2%)
	60°	7.2 (11%)	86 (2.5%)	184 (6.5%)	9.6 (3.7%)
	90°	11.6 (5.7%)	272 (20%)	418 (8.0%)	5.0 (3.0%)
3D-TS-FRC	0°	9.4 (14%)	94 (6.5%)	225 (14%)	3.4 (17%)
	30°	6.7 (3.0%)	65 (8.0%)	176 (6.4%)	9.9 (14%)
	45°	6.5 (5.0%)	61 (5.0%)	162 (1.3%)	11.7 (3.5%)
	60°	9.2 (1.0%)	101 (2.5%)	208 (2.8%)	8.3 (7.3%)
	90°	14.2 (8.2%)	378 (8.0%)	455 (4.9%)	4.3 (2.3%)

**Table 3:** The relative severity of damage mechanisms for changing fibre orientation.

Mat.	Damage Type	Damage Mechanisms	Case-1 (0º)	Case-2 (30º)	Case-3 (45º)	Case-4 (60º)	Case-5 (90º)
3D-TP-FRC	Intralaminar	Fibre breakage	Moderate	None	None	None	Moderate
		Plasticization	Some	Moderate	Significant	Moderate	Some
		Matrix cracking	None	None	Some	None	None
		Kinking	Some	None	None	None	Some
	Interlaminar	Yarn debonding	Some	None	None	Moderate	Moderate
		Yarn re-orientation	None	Moderate	Significant	Moderate	None
		Matrix deformation	Some	Moderate	Significant	Moderate	Some
3D-TS-FRC	Intralaminar	Fibre breakage	Moderate	None	None	None	Significant
		Plasticization	None	None	None	None	None
		Matrix cracking	Moderate	Significant	Significant	Significant	Moderate
		Kinking	Some	None	None	None	Some
	Interlaminar	Yarn debonding	Moderate	Moderate	Moderate	Significant	Some
		Yarn reorientation	None	Moderate	Significant	Moderate	None
		Matrix comp. failure	Some	Moderate	Significant	Moderate	Some

## Data Availability

The presented in this study are available upon request from the corresponding author.
